# What factors contribute towards ambulance on-scene times for suspected stroke patients? An observational study

**DOI:** 10.1177/23969873231163290

**Published:** 2023-03-16

**Authors:** Graham McClelland, Emma Burrow, Abi Alton, Lisa Shaw, Tracy Finch, Chris Price

**Affiliations:** 1 Stroke Research Group, Population Health Sciences Institute, Newcastle University, Newcastle upon Tyne, UK; 2 North East Ambulance Service NHS Foundation Trust, Newcastle upon Tyne, UK; 3 Department of Nursing, Midwifery & Health, Northumbria University, Newcastle upon Tyne, UK

**Keywords:** Stroke, ambulance, time

## Abstract

**Introduction:**

Pre-hospital stroke care focusses on rapid access to specialist stroke units, but UK ambulance data shows increasing pre-hospital times. This study aimed to describe factors contributing towards ambulance on-scene times (OST) for suspected stroke patients and identify targets for a future intervention.

**Patients and methods:**

Ambulance clinicians in North East Ambulance Service were asked to complete a survey after transporting any suspected stroke patients to describe the patient encounter, interventions and timings. Completed surveys were linked with electronic patient care records. Potentially modifiable factors were identified by the study team. Poisson regression analysis quantified the association of selected potentially modifiable factors with OST.

**Results:**

About 2037 suspected stroke patients were conveyed between July and December 2021, resulting in 581 fully completed surveys by 359 different clinicians. The median age of patients was 75 years (interquartile range (IQR) 66–83) and 52% of patients were male. Median OST was 33 min (IQR 26–41). Three potentially modifiable factors were identified as contributors to extended OST. Performing additional advanced neurological assessments added 10% to OST (34 vs 31 min, *p* = 0.008); intravenous cannulation added 13% (35 vs 31 min, *p* = <0.001) and ECGs added 22% (35 vs 28 min, *p* = <0.001).

**Conclusions:**

This study identified three potentially modifiable factors that increased pre-hospital OST with suspected stroke patients. This type of data can be used to target interventions at behaviours that extend pre-hospital OST but which have questionable patient benefit. This approach will be evaluated in a follow up study in the North East of England.

## Introduction

Rapid access to specialist stroke care facilitates receipt of time dependent reperfusion therapies and other urgent treatments (e.g. anticoagulant reversal in haemorrhage). Furthermore, early specialist care minimises complications (e.g. aspiration), and is known to benefit all stroke patients.^1^ Reducing delays between symptom onset and access to specialist stroke units is therefore an important aspect of improving care and patient outcomes.

Approximately two-thirds of acute stroke patients in England are taken to hospital by emergency ambulance^2^ and because ambulance clinicians are unable to offer any specific treatment for stroke, their main role is recognition and rapid transport to appropriate specialist care. The pre-hospital care process includes multiple time segments such as call to dispatch, travel to and from the scene and on-scene time (OST), each of which contribute to the overall call to hospital arrival time. Despite the importance of rapid access to hospital care, in the UK, pre-hospital times (symptom onset to arrival at hospital) have increased in recent years.^3^

Of the pre-hospital time intervals, OST which may be considered the most modifiable, has steadily increased in the past decade from around 20 min in 2011 to 30 min in 2018^4^ and 33 min in 2019.^5^ Other countries report shorter OST, for example, a median of 15 min in America^6^ and a mean of 23 min in Australia.^7^ However, there should be little difference in pre-hospital stroke care between the UK and these countries so UK OST appear extended. UK national ambulance guidelines^8^ make clear recommendation about minimising OST for stroke patients and regional ambulance services have tried to reduce OST (and overall call to hospital times) through clinician education and service wide targets,^9^ but no significant improvement has been reported. In order to improve UK OST, data are required about factors which contribute to the current OST to identify potentially modifiable aspects which could be targeted by an intervention.

## Aim

The aim of this study was to describe factors contributing to ambulance OST for suspected stroke patients and identify potentially modifiable targets for a future intervention.

## Methods

An online survey was used to collect data about the care of suspected stroke patients transported to hospital by ambulance clinicians. Completed surveys were combined with information extracted from the routine ambulance clinical record for each patient. This study is reported using the CHERRIES guidelines.^10^

### Setting

This study was conducted in North East Ambulance Service NHS Foundation Trust (NEAS) which is one of ten regional ambulance services in England. NEAS serves Northumberland, Tyne and Wear, County Durham, Darlington and Teesside serving a population of over 2.7 million and manages over 1.5 million calls per year. NEAS clinicians primarily use the Face Arms Speech test (FAST)^11^ to recognise stroke which is supported by UK national guidelines.^8^

### Survey

A survey was developed to collect data on factors identified from the literature and by discussion within the study team potentially contributing to OST. The survey incorporated aspects from similar work undertaken in Denmark^12^ and previously identified barriers to providing pre-hospital stroke care.^13^ The survey was piloted on a small group (*n* = 5) of NEAS paramedics before being finalised.

The survey was delivered online using SurveyMonkey^14^ and included 20 questions over six pages with 17 compulsory questions and three optional questions. There was no review step when participants completed the survey. See Supplemental Material 1 for the survey questions.

Information about the overall study aim (understanding of factors influencing OST leading to an intervention) was provided at the start of the survey and completion of the survey was taken as consent. Personal identifiable information was held within NEAS as the employing organisation and study sponsor.

Any NEAS clinician who transported a stroke patient to hospital was eligible to complete the survey. Potential participants were notified about the study via information circulated within NEAS via internal newsletters, research webpages and social media. In addition, a clinical record query was used to identify clinicians who transported stroke patients in the previous 24 h, who were emailed a request to complete the survey for the relevant case.

An incentive in the form of a monthly prize draw was offered to increase survey completion rates. For each survey response participants received one entry into a monthly draw to win shopping vouchers.

### Routine clinical records

Data were collected from routine electronic patient care records (EPCRs) to minimise survey length. Completed surveys included NEAS case numbers which were used to identify the corresponding EPCR. Data were manually extracted and combined with survey data in an Excel spreadsheet. Data fields collected from the EPCR are listed in Supplemental Material 2.

### Analysis methods

Average OST was calculated based on the presence or absence of factors extracted from the EPCR and survey. OST was calculated based on the difference between ‘At scene’ and ‘Leave scene’ times.

Data were analysed in three stages:

1. Factors from the EPCR and survey data were descriptively reported against median OST in minutes.
2. Data were examined and discussed within the study team to identify factors which were potentially modifiable and could be candidates for stage three.
3. The impact of the potentially modifiable factors on the OST was analysed using multivariate regression. As the dependent variable (OST) was count data Poisson regression analysis was used.

The IBM Statistical Package for the Social Sciences (SPSS, v24) was used to support data analysis. A *p*-value of 0.05 was used to judge significance.

## Results

### Survey and EPCR data

The survey ran between 7/7/21 and 6/12/21 and was started 1073 times. Excluding incomplete surveys and duplicates resulted in 583 unique surveys completed by 359 different clinicians. The median number of surveys completed by individual clinicians was 1 (IQR 1–2).

After linking surveys with EPCRs two cases were removed due to being unable to establish the OST from the EPCR, therefore 581 cases were included in the analysis. NEAS audit data reports that 2037 stroke cases were seen during the same time period indicating approximately 29% of cases resulted in a completed survey.

OST had a non-normal distribution so times are reported as median with interquartile range (IQR). The median OST was 33 min (IQR 26–41). Only 21% of cases (*n* = 121) met the NEAS target of <25 min OST.

#### Survey data

Data from the surveys are summarised in Table 1 with median OST.

**Table 1.** Data from surveys.

Survey question	Survey answer	*N*	%	Median on-scene time in minutes (IQR)
Was stroke mentioned on the case notes or by control prior to arriving at scene?	Yes	426	73	32 (25–39)
	No	155	27	35 (27–46)
Was stroke your primary diagnosis or one of your differentials?	Primary	429	74	31 (25–39)
	Differential	152	26	38 (29–49)
How certain were you that this patient was having a stroke?	Extremely	152	26	29 (23–35)
	Very	142	24	34 (24–41)
	Somewhat	159	27	35 (28–46)
	Not so	100	17	37 (27–45)
	Not at all	29	5	36 (30–46)
How long after arriving on scene did you start treating the patient as a stroke patient?	Median 5 min (IQR 2–10)	155	27	NR
Was this patient within the stroke acute treatment window?	Yes	363	62	31 (23–39)
	No	218	38	35 (29–46)
How easy was it to establish the last known well time?	NR	2	<1	48 (47–48)
	Very easy	201	35	30 (22–37)
	Easy	173	30	34 (27–42)
	Neutral	57	10	35 (28–43)
	Difficult	99	17	35 (29–49)
	Very difficult	49	8	35 (29–47)
Was the patient FAST positive?	Yes	500	86	33 (26–41)
	No	81	14	35 (29–48)
Who was the primary source of information about the patient?	Patient	184	32	32 (25–39)
	Family	277	48	33 (26–41)
	Bystander	10	2	26 (21–31)
	Carer	68	12	39 (31–46)
	Other	42	7	35 (22–46)
How many people (not including NEAS staff) were on scene?	Median 2 (IQR 1–3)	581	100	NR
FAST	On Scene	559	96	33 (26–41)
	In The Ambulance Before Leaving	139	24	34 (26–42)
	Whilst Travelling	94	16	30 (24–37)
Cranial nerve exam	On Scene	214	37	31 (24–39)
	In The Ambulance Before Leaving	52	9	29 (23–39)
	Whilst Travelling	37	6	31 (28–39)
Other neurological exam	On Scene	256	44	31 (24–38)
	In The Ambulance Before Leaving	68	12	30 (24–39)
	Whilst Travelling	61	10	29 (24–39)
Blood pressure (manual)	On Scene	143	25	33 (26–41)
	In The Ambulance Before Leaving	35	6	35 (26–45)
	Whilst Travelling	15	3	37 (26–47)
Blood pressure (automatic)	On Scene	498	86	34 (27–42)
	In The Ambulance Before Leaving	300	52	32 (25–40)
	Whilst Travelling	231	40	33 (26–41)
ECG	On Scene	275	47	37 (29–46)
	In The Ambulance Before Leaving	157	27	31 (26–39)
	Whilst Travelling	54	9	30 (26–38)
Blood glucose	On Scene	525	90	34 (26–42)
	In The Ambulance Before Leaving	56	10	30 (22–35)
	Whilst Travelling	7	1	41 (27–53)
Intravenous cannulation	On Scene	81	14	38 (29–45)
	In The Ambulance Before Leaving	118	20	34 (29–46)
	Whilst Travelling	42	7	27 (22–35)
Communication with hospital (not pre-alerting)	On Scene	158	27	35 (29–44)
	In The Ambulance Before Leaving	173	30	34 (28–42)
	Whilst Travelling	45	8	29 (22–35)
Pre-alerting	On Scene	47	8	35 (28–44)
	In The Ambulance Before Leaving	184	32	31 (25–39)
	Whilst Travelling	111	19	30 (22–38)
Where was the patient?	Ground floor	362	62	32 (26–39)
	House, upstairs	70	12	37 (29–45)
	Flat not on ground floor	36	6	35 (28–46)
	Nursing or care home	52	9	38 (33–43)
	Other	61	10	27 (21–35)
How was the patient extracted from the location?	Walked	176	30	29 (22–39)
	Carry chair	269	46	35 (28–42)
	Stretcher	136	23	34 (27–43)
Were the patients’ medications easily available?	Yes	463	80	33 (26–41)
	No	78	13	31 (23–42)
	Other	40	7	34 (27–43)
Were blue lights and sirens used to transfer the patient to hospital?	Yes	382	66	31 (25–39)
	No	199	34	35 (29–46)
Barriers to prehospital care (based on Li et al.^13^)	Access	44	8	38 (29–46)
	Patient Assessment and Management	114	20	37 (29–46)
	Communication	149	26	35 (28–42)
	Extrication and Transport	115	20	36 (29–46)
	Patient Refusal	10	2	30 (20–41)
	No barriers	297	51	30 (23–38)

Most patients (86%) were recorded as FAST positive on the survey which closely matches the figure from the survey reported below (87%). The number of patients judged to be in the treatment window closely matches the number of patients where blue lights and sirens were used, and the times align suggesting a link between knowledge of treatment windows and rapid transport which may need further exploration.

The survey identified whether actions happened on scene, in the ambulance, whilst travelling or at multiple points. The general pattern is that most actions were completed on scene and the fewest actions were completed whilst travelling apart from IV cannulation, communication with the hospital and pre-alerting which were most frequently completed in the ambulance. Completion of actions whilst travelling led to the shortest OST in most cases although this would not always be recommended due to safety issues with actions such as IV cannulation whilst travelling and accuracy issues with ECGs.

The survey included questions on patient location and extrication which revealed that most patients (62%) were on the ground floor, which contradicts a common ambulance service belief that all patients are upstairs. When patients weren’t on the ground floor or when they were unable to walk to the ambulance OSTs were extended which makes sense.

#### EPCR data

Data from the EPCRs are summarised in Tables 2 and 3.

**Table 2.** Data from EPCRs (numerical variables).

Variable (EPCR)	Median (IQR)	*N*	%
Patient age (years)	75 (66–83)	581	100
Initial blood glucose (mmol/L)	6.4 (5.4–7.9)	576	99
Initial Glasgow Coma Score (GCS)	15 (14–15)	576	99
Initial heart rate (beats per minute)	80 (71–94)	578	99
Initial systolic blood pressure (mmHg)	125 (134–176)	580	100
Initial National Early Warning Score 2 (NEWS2)	1 (0–3)	533	92

**Table 3.** Data from EPCRs (categorical variables).

Variable (EPCR)	Group	*N*	%	Median on-scene time in minutes (IQR)
Paramedic present	Yes	527	91	33 (26–41)
	No	54	9	33 (27–42)
Gender	Male	303	52	33 (26–41)
	Female	278	48	34 (27–43)
Call category	C1	52	9	34 (26–41)
	C2	503	87	33 (26–41)
	C3	19	3	33 (27–45)
	C4, C5 and Urgent	7	1	37 (20–59)
Receiving ward	Emergency department	387	67	34 (25–42)
	Stroke unit	192	33	33 (27–41)
	Admissions unit	2	<1	20 (17–23)
Seizures	Yes	32	6	38 (29–45)
	No	549	94	33 (26–41)
Vomiting	Yes	49	8	35 (28–44)
	No	532	92	33 (26–41)
Dizziness	Yes	75	13	35 (27–45)
	No	506	87	33 (26–41)
Confusion	Yes	140	24	34 (28–44)
	No	441	76	33 (25–41)
FAST	Positive	505	87	33 (26–41)
	Negative	76	13	37 (29–46)
Prealert	Yes	327	56	30 (24–39)
	No	254	44	35 (29–46)
Barriers to prehospital care (based on Li et al.^13^)	Access	40	7	37 (29–46)
	Assessment and management	118	20	37 (29–45)
	Communication	170	29	35 (28–42)
	Extrication and transport	111	19	37 (29–46)
	Refusal	10	2	30 (20–41)
ECG	Yes − unknown	2	<1	26 (22–29)
	Yes − 3-lead	60	10	30 (22–37)
	Yes − 12-lead	288	50	36 (29–45)
	No	231	40	29 (22–38)
Intravenous cannulation	Yes	185	32	34 (26–41)
	No	396	68	33 (25–42)
Drugs given (including oxygen)	Yes	108	19	37 (29–46)
	No	473	81	32 (25–40)
Fluids given	Yes	5	1	38 (29–46)
	No	576	99	33 (26–41)
COVID concerns	Yes	8	1	36 (28–47)
	No	573	99	33 (26–41)
Healthcare practitioner referral	Yes	20	3	33 (26–49)
	No	561	97	33 (26–41)
Alcohol/recreational drugs	Yes	13	2	42 (29–48)
	No	568	98	33 (26–41)

The demographics and clinical data from the EPCRs are largely as would be expected for a suspected stroke population identified by ambulance clinicians. A large variety of patient past medical histories were recorded on the EPCRs including: hypertension (*n* = 202, 35%), previous stroke (*n* = 130, 22%), diabetes (*n* = 122, 21%), atrial fibrillation (*n* = 91, 16%) and normally fit and well or no recorded medical history (*n* = 39, 6%). The small number of patients with documented COVID concerns (*n* = 8, 1%) is surprising given the timing of the survey.

The presence of any complicating factors (vomiting, seizures, etc) or the completion of any intervention (ECG, drug/fluid administration, etc) increased OST but very few factors were clearly associated with extremes of OST unless the number of patients was small (e.g. use of alcohol/recreational drugs).

### Selection of potentially modifiable factors for a future ambulance service intervention

The data collected from the survey and EPCRs were examined and discussed within the study team to identify factors which were potentially modifiable and could be candidates for an intervention aimed at reducing OST.

The following factors were identified as potential intervention targets: pre-alerting, ECGs, administering drugs; administering fluids, clinician certainty, clinician knowledge of treatment windows, cranial nerve exams, other neurological exams, intravenous cannulation, communication with the hospital, use of blue lights and sirens.

These factors were considered against local and national guidelines, impact on OST, frequency and ease of targeting with an intervention.

Communication with hospital, pre-alerting and use of light and sirens were excluded due to their inclusion in local and national stroke guidelines. The administration of drugs and/or fluids were assumed to be appropriate responses to the patients’ presentation so were also excluded. Subjective factors that may be difficult to measure in an intervention were excluded (e.g. certainty in stroke diagnosis, knowledge of treatment windows). Cranial nerve exam and other neurological exam factors were combined into a single advanced assessment factor representing anything beyond the basic FAST test.

Three factors were selected as potential intervention targets for further consideration: advanced assessments, ECGs and IV cannulation.

### Impact of potential intervention targets on OST

Timing data from the EPCR was combined with usage data from the survey to explore the impact of the three targets on OST. Some factors were recorded in both the survey and the EPCR however survey data were used as advanced assessments were not routinely recorded in the EPCR and because survey data allowed interventions that happened on-scene to be isolated from those that happened whilst travelling which was not possible using EPCR data.

The impact of the three potential intervention targets on OST was analysed using Poisson regression analysis and is reported in Table 4. The analysis adjusted for all factors included and a chi-square parameter was added to account for over dispersion.

**Table 4.** Impact of potential intervention targets on OST.

	Number (%)	Median time inminutes(IQR)	Rate ratio (Exp (B) 95% CI)	*p*-Value
All cases	581 (100)	33 (26–41)	–	–
Advanced assessment+	368 (63)	34 (27–44)	1.097 (1.025–1.175)	0.008
Advanced assessment−	213 (37)	31 (23–38)	1	
ECG+	415 (71)	35 (28–44)	1.223 (1.134–1.320)	<0.001
ECG−	166 (29)	28 (22–35)	1	
Intravenous cannulation+	198 (34)	35 (29–46)	1.132 (1.060–1.208)	<0.001
Intravenous cannulation−	383 (66)	31 (25–39)	1	

Advanced assessment: cranial nerve exam and/or other neuro exam; ECG: any recording of an electrocardiogram.

The regression analysis shows that all three potential targets significantly impacted on OST. Advanced assessments increased OST by 10% (rate ratio 1.097, *p* = 0.008), intravenous cannulation increased OST by 13% (rate ratio 1.132, *p* = <0.001) and ECGs increased OST by 22% (rate ratio 1.223, *p* = <0.001).

Further analysis of the data on these three potential targets showed that the majority (84%) of advanced assessments were completed in patients who were FAST positive. Most ECGs were completed on scene as opposed to in the ambulance or whilst travelling (Table 1) and 12-lead ECGs rather than 3-lead ECGs were the main contributors to longer OST. Only 23 (12%) patients who were cannulated had any fluids (*n* = 5, 3%) or drugs (*n* = 18, 9%) administered via the cannula in the pre-hospital setting.

## Discussion

This study describes the contribution of key clinical factors on OST for suspected stroke patients in a UK ambulance service. By combining information collected from clinicians and clinical records three factors (advanced assessments, ECG, intravenous cannulation) were identified which increase OST and which may be targets for a future intervention

The characteristics of the patient population described in this study are like other UK pre-hospital stroke studies reported elsewhere.^15–17^ The median OST was 33 min which is in line with previously reported times from the same region,^4,5^ is similar to OSTs reported by other UK services^18,19^ but higher than international comparators.^6,7,20^ Ambulance OST has been reported for other time critical conditions which are consistently lower than UK OST for stroke, median 16 min (IQR 16–20) reported for acute chest pain patients in the United States,^21^ median 17 min (IQR 13–23) for critical patient in Kawasaki, Japan,^22^ mean 16–18 min for trauma patients in the United States^23^ and median 17–22 min for high priority patients in Denmark.^24^

The findings of this study are in line with previous studies and national recommendations. Extending OST to cannulate and perform ECGs on stroke patients are both advised against in UK ambulance guidelines.^8^ Cannulation and ECGs have been shown to extend OST^12,20^ and ECGs have been associated with extended OST and worse patient outcomes.^17^ 3-lead ECGs take little time and can be used to monitor patients and identify AF which is a recognised stroke risk factor whereas 12-lead ECGs appear to be the main contributor to extended OST in this study. IV cannulation may be necessary to administer fluids or medication but 88% of cannulas were not used for pre-hospital medication or fluids in this study. The impact of pre-hospital cannulation or ECGs on hospital practice and overall time from ambulance call to definitive treatment could be investigated in a further study.

A 2020 study by Li et al.^20^ highlighted IV cannulation, ECGs and blood glucose as pre-hospital interventions that extended OST and recommended doing interventions whilst travelling as opposed to on-scene. Whilst IV cannulation and ECGs were also identified as potentially modifiable behaviours in this study blood glucose is recommended in UK practice to identify hypoglycaemia as a stroke mimic condition that could potentially be addressed at scene therefore this was not considered as a potential target for changing behaviour or reducing OST.

Like this study Drenck et al.^12^ reported that ECGs and IV cannulation extended OST and suggested they could be done during transport or in hospital to reduce OST. The quality of communication was also highlighted by Drenck et al.^12^ as influencing OST, whilst this was not explored in this study communication with the hospital whilst travelling, as opposed to on-scene, appears to reduce the OST but relies on the ambulance crew being able to decide on the destination before leaving. A previous study in NEAS showed that different methods of communication between ambulance and hospital influence the OST.^25^

There are many pre-hospital stroke assessment tools described in the literature^26^ and the benefits of ambulance clinicians identifying more stroke patients is self-evident, however, the use of advanced neurological assessments in patients who are largely (86%–87%) FAST positive needs to be considered if it unnecessarily extends OST and does not change ambulance clinician behaviour or patient outcomes.

The population described includes suspected stroke patients based on ambulance data so does not include final diagnoses, hospital actions or patient outcomes which would be necessary to truly understand the impact of actions and time spent in the pre-hospital setting. Data were collected during the second year of the COVID-19 pandemic so any actions, behaviour and times reported need to bear this context in mind although the small number of patients with COVID concerns recorded may indicate this is not a major concern.

Previous interventions aimed at improving the efficiency of emergency stroke care have rarely reported significant savings in pre-hospital OST, have mostly used education as the mechanism of action and haven’t targeted individual factors such as those identified in this study.^27^ Using data on factors contributing to extended OST such as presented here may allow interventions to target specific clinician behaviours and actions and reduce pre-hospital OST which should then benefit patients.

## Strengths and limitations

This study includes data on a wide range of factors that may influence pre-hospital OST for stroke patients. The data were collected from two sources very close to the time of the event so should represent an accurate record of what happened. The survey allowed collection of information which was not available from clinical records alone, such as the patient being upstairs. Combining clinician witness information with clinical records is a novel approach which could be useful for examining other ambulance scenarios. The combination of retrospective routine data and prospective survey data addresses some of the limitations of either dataset in isolation.

Limitations include that only 29% of suspected stroke cases were included, surveys were completed by a self-selecting population so may not be representative of all practitioners and data were collected for a limited time in only one region.

The regression candidate variables were selected based on a series of subjective decisions by the study team supported by descriptive data. Statistical analyses of the whole dataset, possibly using different methods such as machine learning, may identify other factors that have an influence OST but may not be modifiable.

## Conclusions

This study used surveys linked with clinical records to identify three potentially modifiable factors that increased pre-hospital OST with suspected stroke patients. The authors recommend that this type of data be used to target interventions at behaviours that extend pre-hospital OST but which have questionable patient benefit. This approach will be evaluated in a follow up study in the North East of England.

## Supplemental Material

sj-docx-1-eso-10.1177_23969873231163290 – Supplemental material for What factors contribute towards ambulance on-scene times for suspected stroke patients? An observational study Click here for additional data file. Supplemental material, sj-docx-1-eso-10.1177_23969873231163290 for What factors contribute towards ambulance on-scene times for suspected stroke patients? An observational study by Graham McClelland, Emma Burrow, Abi Alton, Lisa Shaw, Tracy Finch and Chris Price in European Stroke Journal

sj-docx-2-eso-10.1177_23969873231163290 – Supplemental material for What factors contribute towards ambulance on-scene times for suspected stroke patients? An observational study Click here for additional data file. Supplemental material, sj-docx-2-eso-10.1177_23969873231163290 for What factors contribute towards ambulance on-scene times for suspected stroke patients? An observational study by Graham McClelland, Emma Burrow, Abi Alton, Lisa Shaw, Tracy Finch and Chris Price in European Stroke Journal
