# Supplementary material for: What factors contribute towards ambulance on-scene times for suspected stroke patients? An observational study
Source: Eur Stroke J. 2023 Mar 16;8(2):492–500. doi: 10.1177/23969873231163290 (PMC10334177; doi:10.1177/23969873231163290)
Supplement: sj-docx-1-eso-10.1177_23969873231163290 – Supplemental material for What factors contribute towards ambulance on-scene times for suspected stroke patients? An observational study [file sj-docx-1-eso-10.1177_23969873231163290.docx]

**Supplementary material 1 - Stroke Time Survey**

**INTRODUCTION PAGE**

**The Pre-hospital stroke time study**

Minutes matter for acute stroke patients. The untreated acute ischaemic stroke patient loses 1.9 million neurons per minute so rapid access to treatment is vital. Delays of minutes impact on patient outcomes due to the narrow therapeutic window and the decreasing availability and effectiveness of treatment over time.

This study will explore how ambulance clinicians spend their time with suspected stroke patients in order to inform future work to minimise the time spent in the pre-hospital phase. The study is being completed as part of a post-doctoral fellowship by the lead researcher Dr Graham McClelland.

This survey asks about your actions, decision making and factors that may have influenced the time spent with the stroke patient you recently handed over at hospital. This data is being collected as part of the research project described above.

All survey results will be anonymised. Case ID is required so timings and other data can be extracted from the EPCR. Clinician ID number is required so you can be entered into the prize draw for each survey completed. At the end of the survey you have the option of volunteering to take part in an interview with a researcher from Newcastle University to further explore this topic.

Please contact Graham McClelland [graham.mcclelland@neas.nhs.uk](mailto:graham.mcclelland@neas.nhs.uk) if you have any questions or concerns about the survey.

Completion of this survey is taken to indicate consent to be included in this study.

**SURVEY QUESTIONS**

Case ID: (XXXXXXXX)

Clinician ID number: (XXXXXXXX)

Was stroke mentioned on the terrafix or by control prior to arriving at scene? (yes/no)

If above = No – when did you decide to treat the patient as a stroke (approx. minutes after arriving on scene)?

Was stroke your primary diagnosis or one of your differentials? (primary/differential)

How certain were you that this patient was having a stroke? (1-5 LIKERT SCALE, 1= very uncertain, 3= certain, 5= very certain)

Was this patient within the stroke acute treatment window? (yes/no)

Was the patient FAST+ (yes/no)

If above = yes – F, A, S (pick one or multiple)

If above = no – What made you suspect stroke? (freetext)

Who was the primary source of information about the patient? (patient/family/carer/bystander/other)

Number of people (family/bystanders/carers) on scene in addition to the patient? (number)

| **Actions performed** | | | |
| --- | --- | --- | --- |
|  | **On scene** | **In the ambulance before leaving scene** | **Whilst travelling** |
| FAST |  |  |  |
| Cranial nerve exam |  |  |  |
| Other neuro exam |  |  |  |
| BP |  |  |  |
| ECG |  |  |  |
| BM |  |  |  |
| IV |  |  |  |
| Communication with hospital |  |  |  |

Tick multiple boxes if actions happened at more than one point.

Where was the patient? (ground floor house/bungalow/flat, house upstairs, flat upstairs, nursing/care home, other)

How was the patient extracted from the location? (walked, carry chair, stretcher)

Were the patients’ medications easily available? (yes/no)

Did you have to communicate with the hospital before deciding where to take the patient? (yes/no)

Did you place a pre-alert (yes via control/yes direct to hospital/no)

Were blue lights and sirens used to transfer the patient to hospital (yes/no)

Did you have difficulty with any of the factors below which extended your time with the patient? (access, patient assessment and management, communication, patient extrication and transport, patient refusal)

Are you willing to interviewed about pre-hospital stroke care (not this specific case) at a later date by a researcher from Newcastle University in order to explore this topic further? (yes/no)

If above = yes – Name and contact details.

Thank you. Either Graham McClelland or the researcher will be in touch with more information about the interview.
