# Supplementary material for: What factors contribute towards ambulance on-scene times for suspected stroke patients? An observational study
Source: Eur Stroke J. 2023 Mar 16;8(2):492–500. doi: 10.1177/23969873231163290 (PMC10334177; doi:10.1177/23969873231163290)
Supplement: sj-docx-2-eso-10.1177_23969873231163290 – Supplemental material for What factors contribute towards ambulance on-scene times for suspected stroke patients? An observational study [file sj-docx-2-eso-10.1177_23969873231163290.docx]

**Supplementary material 2 – data fields collected from NEAS EPCRs**

Date

Call time

At scene time

At patient time

Leave scene time

At hospital time

Receiving hospital

Receiving ward/unit

Seizures

Vomiting

Dizziness

Confusion

FAST+ face

FAST+ arms

FAST+ legs

FAST+ speech

Onset/LKW

Prealert

Difficult access

Difficult patient assessment and management

Difficult communication

Difficult extrication and transport

Difficult patient refusal

Initial blood glucose

Initial GCS

Initial AcVPU

Initial HR

Initial SBP

NEWS2

PMH

ECG

ECG rhythm recorded

IV access

IV attempts

Any drugs given (inc O2)

Any fluids given

No of impressions recorded

COVID concern

Healthcare practitioner referral

Alcohol/recreational drugs
